# Abnormal Anatomical Connectivity between the Amygdala and Orbitofrontal Cortex in Conduct Disorder

**DOI:** 10.1371/journal.pone.0048789

**Published:** 2012-11-07

**Authors:** Luca Passamonti, Graeme Fairchild, Alex Fornito, Ian M. Goodyer, Ian Nimmo-Smith, Cindy C. Hagan, Andrew J. Calder

**Affiliations:** 1 Consiglio Nazionale delle Ricerche, Unità di Ricerca Neuroimmagini, Catanzaro, Italy; 2 Medical Research Council, Cognition and Brain Sciences Unit, Cambridge, United Kingdom; 3 Department of Psychiatry, University of Cambridge, Cambridge, United Kingdom; 4 School of Psychology, University of Southampton, Southampton, United Kingdom; 5 Melbourne Neuropsychiatry Centre, Department of Psychiatry, University of Melbourne, Victoria, Australia; 6 Cambridge and Peterborough National Health Service Foundation Trust, Cambridge, United Kingdom; University of South Florida, United States of America

## Abstract

**Objective:**

Previous research suggested that structural and functional abnormalities within the amygdala and orbitofrontal cortex contribute to the pathophysiology of Conduct Disorder (CD). Here, we investigated whether the integrity of the white-matter pathways connecting these regions is abnormal and thus may represent a putative neurobiological marker for CD.

**Methods:**

Diffusion Tensor Imaging (DTI) was used to investigate white-matter microstructural integrity in male adolescents with childhood-onset CD, compared with healthy controls matched in age, sex, intelligence, and socioeconomic status. Two approaches were employed to analyze DTI data: voxel-based morphometry of fractional anisotropy (FA), an index of white-matter integrity, and virtual dissection of white-matter pathways using tractography.

**Results:**

Adolescents with CD displayed higher FA within the right external capsule relative to controls (T = 6.08, P<0.05, Family-Wise Error, whole-brain correction). Tractography analyses showed that FA values within the uncinate fascicle (connecting the amygdala and orbitofrontal cortex) were abnormally increased in individuals with CD relative to controls. This was in contrast with the inferior frontal-occipital fascicle, which showed no significant group differences in FA. The finding of increased FA in the uncinate fascicle remained significant when factoring out the contribution of attention-deficit/hyperactivity disorder symptoms. There were no group differences in the number of streamlines in either of these anatomical tracts.

**Conclusions:**

These results provide evidence that CD is associated with white-matter microstructural abnormalities in the anatomical tract that connects the amygdala and orbitofrontal cortex, the uncinate fascicle. These results implicate abnormal maturation of white-matter pathways which are fundamental in the regulation of emotional behavior in CD.

## Introduction

Conduct Disorder (CD) is a psychiatric condition characterized by a pervasive pattern of aggressive and antisocial behavior [1]. This condition is more common in males compared with females [2] and represents a major public health problem, not only because youths with this disorder may inflict serious physical and psychological harm to others, but because they are also at increased risk for self-injury, major depression, substance abuse, and death by homicide or suicide [3]–[5].

Accumulating evidence suggests that CD has a neurobiological basis and involves brain regions implicated in emotion processing [6]–[7]. Consistent with this, individuals with CD show impaired fear conditioning and recognition of facial expressions, and reduced startle magnitudes [8]–[9]. Since these functions have been linked to the amygdala and prefrontal cortex [10]–[14], it is of note that functional magnetic resonance imaging (fMRI) studies have shown that, relative to healthy controls, male adolescents with CD display dysfunctional neural responses in these regions when viewing emotional facial expressions [6] or emotional images [15]–[16]. In addition, studies of children with high levels of callous-unemotional (CU) traits, a constellation of personality traits proposed as a risk factor for CD and psychopathy, showed reduced amygdala activity to fearful faces and decreased functional connectivity between the amygdala and ventromedial prefrontal cortex [17]–[18]. In accord with these findings, structural MRI studies have revealed reduced gray matter volumes within the amygdala, anterior insula, and orbitofrontal cortex (OFC) in adolescents with CD relative to healthy controls [7], [19]–[20]. In contrast, a recent study investigating children with CU traits found increased gray matter concentration in medial OFC and anterior cingulate cortex, suggesting that distinct brain abnormalities may be present across different stages of development [21] or that CD with and without CU traits may be associated with qualitatively different brain abnormalities [22]. Converging evidence therefore supports a key role for both the amygdala and prefrontal cortex regions in the pathophysiology of CD and CU traits. In the current study, we investigate whether adolescents with CD show abnormalities in the white-matter pathways that connect these two regions.

Recent evidence suggests that the uncinate fascicle (UF), the major bundle of fibers connecting the amygdala and adjacent anterior temporal lobe regions to the OFC, shows microstructural abnormalities in adults with psychopathy [23]–[24] and in youths with CD [25]. Consequently, we hypothesized that white-matter abnormalities in the UF would be observed in individuals with CD. To test our prediction, we applied two different but complementary approaches to the analysis of white-matter fractional anisotropy (FA), measured using Diffusion Tensor Imaging (DTI). FA provides an indirect measure of the white-matter microstructure, quantifying changes in both myelination and axonal organization to index overall fiber integrity [26]. FA values range from 0 (perfectly isotropic or non-directional diffusion) to 1 (completely anisotropic or directional diffusion) and are derived from the combination of two measures, axial (eigenvalue λ1) and radial diffusivity (eigenvalues λ2 and λ3) – the rates of diffusion along, versus across, fiber tracts, respectively [26].

We first used whole-brain, voxel-wise comparisons to identify, in a regionally-unbiased manner, pathways in which male adolescents with CD showed altered white-matter integrity relative to healthy controls. We followed up these whole-brain analyses by using tractography to ‘dissect’ the affected pathways [27] and to determine the relative contribution of specific anatomical tracts to the observed group differences in FA.

## Materials and Methods

### Participants

Thirteen male adolescents with CD were recruited from schools, pupil referral units and the Cambridge Youth Offending Service. Exclusion criteria included: full-scale IQ<85, as estimated using the Wechsler Abbreviated Scale of Intelligence (WASI) [28], the presence of a pervasive developmental disorder (e.g., autism) or chronic physical illness. A well-matched group of 13 male healthy controls (no lifetime history of CD/Oppositional Defiant Disorder (ODD) and no current psychiatric illness) were recruited from schools and colleges. To equate groups for IQ, controls with full-scale IQ>115 were excluded.

Participants were assessed for psychiatric disorders based on DSM-IV criteria [1]. Current and lifetime disorders assessed were CD, Oppositional Defiant Disorder (ODD), Attention-Deficit/Hyperactivity Disorder (ADHD), Major Depressive Disorder, Generalized Anxiety Disorder, Obsessive Compulsive Disorder, Post-Traumatic Stress Disorder, and Substance Dependence using the Schedule for Affective Disorders and Schizophrenia for School-age Children-Present and Lifetime Version (K-SADS-PL) [29]. All CD subjects fulfilled criteria for childhood-onset CD, i.e., they reported that at least one symptom of CD was present prior to age 10. Self-reported callous-unemotional and overall psychopathic traits were assessed using the Callous-Unemotional dimension subscale and the total score on the Youth Psychopathic traits Inventory (YPI), respectively [30]. The Spielberger State-Trait Anxiety Inventory was used to assess anxiety [31].

A potential confound when studying neurobiological factors underlying CD is the high co-morbidity with ADHD [32], another common neurodevelopmental condition that may obscure or alter specific effects associated with CD. To limit this problem, all participants were recruited from the community rather than clinics, given the reduced prevalence of concurrent co-morbid illnesses reported in the former setting [33]. In addition, detailed information about ADHD symptoms was obtained using the K-SADS-PL and included as a covariate of no interest in additional analyses controlling for ADHD symptoms.

The study was approved by the Suffolk National Health Service Research Ethics Committee and written informed consent was obtained from all participants. We also obtained written informed consent from parents if the participant was under the age of 18.

### Imaging Protocol

MRI scanning was performed on a 3-Tesla Siemens Tim Trio with a head coil gradient set at the Medical Research Council, Cognition and Brain Sciences Unit. DTI data were acquired using an echo planar imaging (EPI) sequence covering the whole brain (TE/TR = 93/6500 ms, bandwidth = 1396 Hz/Px, field of view (FOV) 230 mm; 48 axial slices, 2.5 mm slice thickness). Diffusion weighted images were sensitized for diffusion along 64 different directions with a b-value of 1000 s/mm^2^. An additional, separate T2-weighted (i.e. b = 0) volume was also acquired.

### Voxel-based Diffusion Tensor Imaging (VB-DTI) Analysis

Eddy-current distortions and head movements during scanning were corrected by rigid-body registration of the diffusion-weighted images onto the subject-specific T2-weighted EPI image using algorithms implemented in the FSL software package (www.fmrib.ox.ac.uk/fsl). Maps of fractional anisotropy (FA), eigenvalues (λ1 or axial diffusivity, λ2 and λ3 or radial diffusivity) and apparent diffusion coefficient (ADC) were then generated for each voxel using the Diffusion Toolkit (http://trackvis.org/dtk/), according to the method described by Basser and Pierpaoli [34]. Next, FA images were spatially normalized onto the T2-weighted template from SPM (www.fil.ion.ucl.ac.uk/spm) and then smoothed with an 8 mm full-width at half-maximum (FWHM) isotropic Gaussian kernel.

Individual FA maps were entered into a second-level two-sample t-test for a voxel-based statistical comparison of regional diffusion differences between adolescents/young adults with CD and healthy controls. To remove potential confounding influences of ADHD, the analyses were repeated including lifetime/ever ADHD symptoms as a covariate of no interest. The significance level was set at P<0.05, corrected for the white matter volume across the entire brain. Correction for the entire white-matter volume was performed by using a whole-brain white-matter mask created with Pick_Atlas (www.fmri.wfubmc.edu/cms/software).

### Fiber Tracking DTI

To further characterize our findings, we followed up voxel-wise comparisons with tractographic analyses using Diffusion Toolkit and Trackvis (www.trackvis.org). After reconstructing and identifying the orientations of maximum diffusion at each voxel location, DTI tractography was performed by seeding every voxel and proceeding in each identified direction of maximum diffusion according to the tracking Fiber Assignment by Continuous Tracking (FACT) procedure described by Basser [35]. Seeding every voxel in this manner helps reduce biases associated with selecting specific seed-points for fiber tracking and mitigates tracking errors caused by fibers crossing within a single voxel. The reconstructed tracts were then graphically rendered to facilitate subsequent regions of interest (ROI) analyses (see below). To minimise reconstruction of spurious pathways, tracts were terminated if the angle of maximum diffusion exceeded 35 degrees.

Since whole-brain DTI analyses (see voxel-based diffusion tensor imaging (VB-DTI) section below) identified increased FA values between individuals with CD and controls within the external capsule, and given prior findings in adult psychopaths [23], we focused our tractography analyses on the two major anatomical tracts that pass through this white-matter area – the uncinate fascicle (UF) and the inferior frontal-occipital fascicle (IFOF). The trajectories of each tract were reconstructed using an approach that involved ‘dissecting’ these fascicles with a combination of different ROIs [23]. Tracts were isolated in all subjects by one of the authors (LP) who was blind to diagnosis and in accordance with an approach previously described [23], [27]. Briefly, three ROIs were created as follows: a ‘temporal lobe’ ROI within the anterior temporal lobe (from MNI: z from −14 to −20); an ‘occipital lobe’ ROI delineated around the occipital lobe on approximately 10 contiguous axial slices (from MNI: z from −4 to 4), and an ‘external capsule’ ROI which included the white matter of the anterior floor of the external/extreme capsule. Only tracts passing through the temporal lobe and external capsule ROI (UF) or passing through the occipital lobe and the external capsule ROI (IFOF) were retained for further analyses. After isolating the UF and IFOF in each subject in this manner, we computed the mean FA, the mean eigenvalues (λ1, λ2, λ3), the apparent diffusion coefficient (ADC) and the average number of streamlines (SLs) per tract and analysed these values using an ANCOVA model that included the subject-specific ROI volume of each tract (i.e., the number of voxels) as covariates of no interest. A further ANCOVA included lifetime ADHD symptoms in addition to the ROI volumes to account for any contribution of ADHD.

## Results

### Participants

**Table 1** summarizes the demographic and clinical characteristics of all participants included in the DTI analyses and reports statistics for the differences in the psychometric variables between individuals with CD and healthy controls. The groups were matched in age, sex, estimated full-scale IQ, and socioeconomic status. As expected, individuals with CD scored higher than controls on current and lifetime/ever CD symptoms, on aggressive CD symptoms, on ADHD symptoms, and on overall psychopathic and callous-unemotional traits. There were no differences in state or trait anxiety between the groups.

**Table 1:** Characteristics of the participants included in the diffusion tensor imaging analyses.

*Measure*	*CD (n = 13)*		*CONTROLS (n = 13)*		
	Mean	SD	Mean	SD	*T, P*
**Age (years)**	18.4	0.8	18.6	0.9	T = −0.7, P = 0.47
**Estimated full scale IQ**	95.4	6.1	98.7	8.5	T = −1.1, P = 0.27
**Psychopathic traits (YPI total)**	2.6	0.3	1.9	0.2	T = 4.5, P<0.001
**YPI callous-unemotional subscale**	0.7	0.1	0.6	0.1	T = 2.7, P = 0.011
**Lifetime/ever CD Symptoms**	10.0	1.2	0.3	0.6	T = 18, P<0.001
**Current CD Symptoms**	5.4	2.5	0	0	T = 7.7, P<0.001
**Aggressive CD Symptoms**	3.7	0.9	0.1	0.3	T = 8.6, P<0.001
**State Anxiety (STAI)**	29.6	6.5	29.5	3.9	T = 0.5, P = 0.95
**Trait Anxiety (STAI)**	40.0	9.3	35.4	5.3	T = 1.4, P = 0.14
**Current ADHD Symptoms**	6.3	4.4	1.2	1.5	T = 3.8, P<0.001
**Lifetime ADHD Symptoms**	8.3	3.8	2.0	2.2	T = 5.8, P<0.001
**ACORN socioeconomic status:**	**N**	**%**	**N**	**%**	***χ^2^ (exact)***
Wealthy achievers (1)	0	0	3	23.1	P = 0.2
Urban prosperity (2)	1	7.7	3	23.1	
Comfortably off (3)	5	38.5	2	15.4	
Moderate means (4)	2	15.3	0	0	
Hard-pressed (5)	5	38.5	5	38.4	

Key: SD, Standard Deviation; IQ, intelligence quotient; YPI, Youth Psychopathic traits Inventory; CD, Conduct Disorder; STAI, Spielberger State-Trait Anxiety Inventory; ADHD, Attention-Deficit/Hyperactivity Disorder.

### Voxel-based Diffusion Tensor Imaging (VB-DTI)

FA values reflect the degree of organization and microstructural integrity of the white-matter bundles.

**Figure 1:**
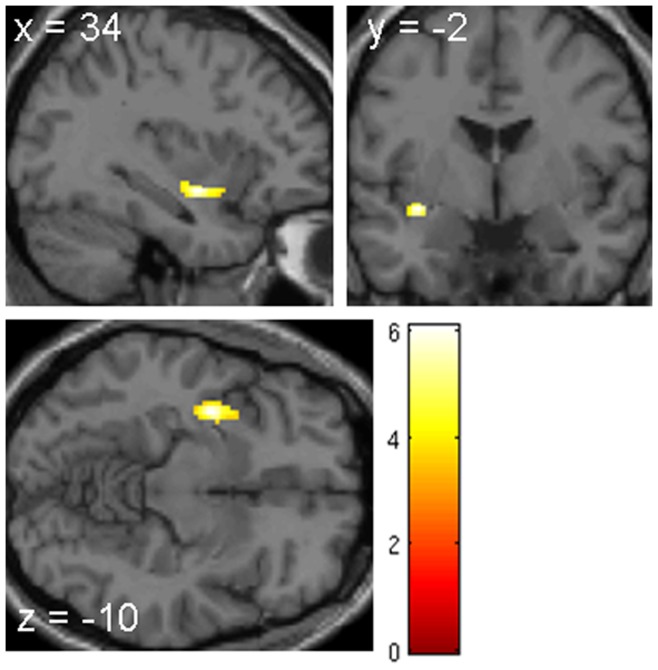
Voxel based whole-brain Diffusion Tensor Imaging analysis. The right external capsule was the only white matter area that emerged as significant when comparing FA maps from individuals with CD relative to age- and IQ-matched healthy controls (two-sample t test, P<0.05, corrected for the entire volume of the white-matter in the whole brain). The CD group showed increased FA values in this region relative to the healthy control group. MNI: Montreal Neurological Institute (x, y, z) coordinates. The color bar ranging from red (bottom) to yellow (top) represents T statistics.

Compared to healthy controls, participants with CD showed significantly higher FA values in a cluster of voxels within the right ventral external capsule (MNI coordinates of the local maxima, x = 34, y = −2, z = −10, T = 6.08, P<0.05, Family-Wise Error (FWE) corrected for the whole white-matter volume) (**Figure 1**). No other regions were identified at this threshold. However, at a lower threshold (P = 0.001, uncorrected), the ventral external capsule on the left side also showed greater FA values in participants with CD relative to controls. The inverse comparison between groups (i.e., Controls > CD) revealed no supra-threshold voxels. Repeating the analyses with lifetime ADHD symptoms as a covariate of no interest also identified the right ventral external capsule (P<0.05, FWE corrected for the whole white-matter volume) but no other regions.

### DTI Tractography

**Figure 2:**
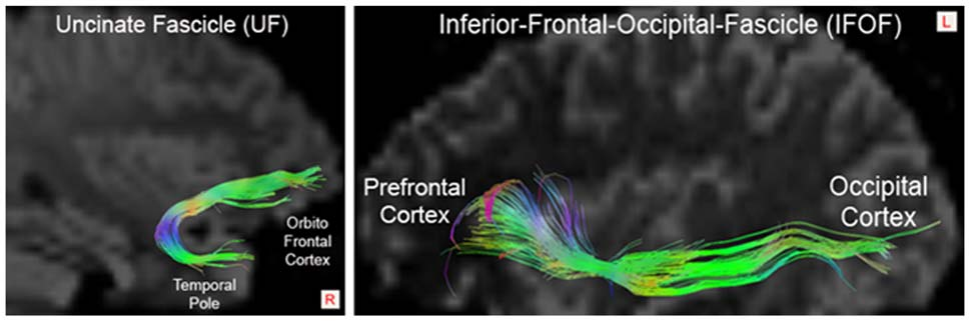
Examples of reconstructions of the right Uncinate Fascicle (left panel) or the left Inferior Frontal-Occipital Fascicle (right panel) pathways in two individual subjects.

**Figure 3:**
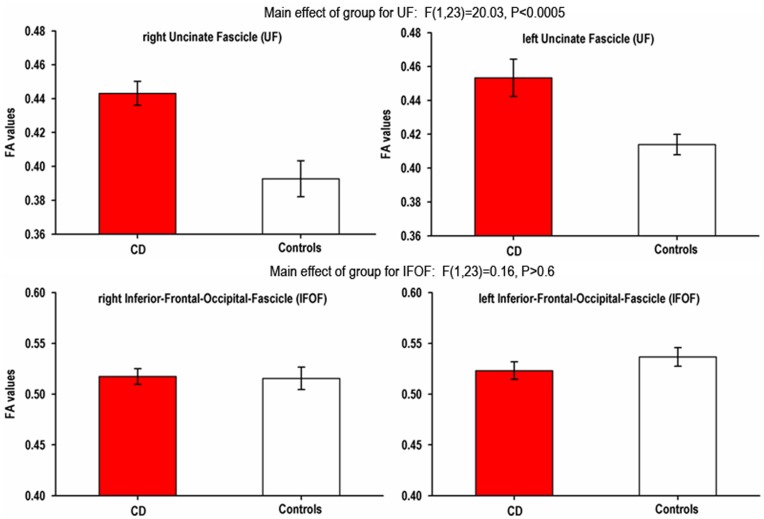
Plots of the fractional anisotropy (FA) values within the bilateral Uncinate Fascicle and the Inferior-Frontal-Occipital Fascicle in individuals with Conduct Disorder (CD) and healthy controls as obtained from tractography analyses. Error bars represent the standard error of the mean.

DTI tractography was used to disentangle the relative contribution of different fascicles constituting the white-matter areas that showed increased FA in subjects with CD, relative to controls, in the whole-brain analyses. Since a cluster of voxels within the right ventral external capsule displayed significant differences in FA values between controls and individuals with CD, we sought to differentiate the contribution of the two anatomical pathways known to pass through the external capsule (i.e., the UF and IFOF). As shown in the examples from individual subjects (**Figure 2**), the fiber tracking procedure correctly delineated both the UF and the IFOF. The UF is a bundle of fibers that originates in the antero-medial temporal lobe (including the amygdala) and passes through the ventral section of the external capsule *en route* to the orbitofrontal and ventromedial prefrontal cortices. Although the IFOF also passes through the external capsule, it connects the occipital lobe with the OFC. On approaching anterior brain regions, fibers of the IFOF enter the external capsule dorsally with respect to the UF and terminate within the lateral parts of the OFC. The external capsule is a crucial white-matter area in which bundles of fibers connecting different brain regions converge; hence, it is important to distinguish whether the UF and/or the IFOF contributed to the group effect revealed by whole-brain VB-DTI analysis. To address this, the mean FA value, the total number of fibers (streamlines), the mean λ1, λ2, λ3 eigenvalues and the ADC mean values *per tract* were calculated in each subject and entered into separate three-way ANCOVAs exploring the effects of tract (UF and IFOF; repeated measure), hemisphere (left and right; repeated measure) and group (CD and control participants; between- subjects factor). **Figure 3**, **Table 2** and **Tables S1, S2, S3, S4, S5, S6** report a summary of these results including the mean FA values, the average number of streamlines, the mean λ1, λ2, λ3 eigenvalues and the mean ADC as reconstructed in the UF and IFOF of individuals with CD and controls. Subject-specific ROI volumes used to construct each tract were included as covariates of no interest in each ANCOVA to factor out any potential contribution they might make to the results. We also repeated the analyses after including lifetime ADHD symptoms as a between-subject covariate of no interest, to ensure that any between-group differences were not attributable to comorbid ADHD symptoms.

The three-way ANCOVA exploring FA values revealed a significant effect of group, F(1,23) = 5.89, P<0.05, which was qualified by a significant group by tract interaction, F(1,23) = 8.67, P<0.01. Further ANCOVAs exploring the effects of group and hemisphere for each tract showed a highly significant effect of group for the UF, F(1,23) = 20.03, P<0.0005, and no corresponding effect for the IFOF, F(1,23) = 0.16, P>0.6. The former group effect was driven by higher FA values in the CD group relative to healthy controls. None of these effects were qualified by a significant interaction with hemisphere (P values>0.1), indicating that the effect of group for the right UF did not significantly differ from the left UF. However, significant overall effects of hemisphere were found for both the 3-way ANCOVA, F(1,23) = 9.94, p<0.005, and each of the two-way ANCOVAs investigating the UF, F(1,23) = 5.83, P<0.05, and IFOF, F(1,23) = 5.62, P<0.05, suggesting that there were differences in the FA of tracts in the two hemispheres, irrespective of group status.

A corresponding three-way ANCOVA exploring FA values and including lifetime/ever ADHD symptoms as an additional covariate showed a similar pattern of results. Note that ADHD symptoms are a between-subject, individual differences measure and therefore do not modulate the within-subject, repeated measures effects or interactions involving within-subjects variables in the ANCOVAs (see **Table S2**). Hence, the group by tract interaction remained unchanged, F(1,23) = 8.67, P<0.01, while the effect of group for the UF remained significant, F(1,22) = 5.11, P = 0.03, although it was of borderline significance (P = 0.06) when correcting for multiple comparisons using the Bonferroni procedure. Consistent with the previous analysis, the main effect of group for the IFOF was not significant, F<1. As would be expected, the effects of hemisphere for the three-way ANCOVA, and analyses of the UF and IFOF remained as reported above.

A second analysis investigated differences in the streamlines using a three-way ANCOVA examining the effects listed above and including subject-specific ROI volumes for each tract as covariates. This showed no significant effects of group, hemisphere, or tract, or interactions among these factors (P values>0.1). Similarly, the ANCOVA including both ROI volumes and lifetime/ever ADHD symptoms as covariates also found no significant effects or interactions with the same factors (Ps>0.1). Thus, consistent with a recent study of adult psychopaths [23], group differences or interactions with group were restricted to FA values and were not found for the number of streamlines. When analysing which of the axial (λ1 eigenvalues) or radial diffusivity (λ2, λ3 eigenvalues) measures was contributing to the main effect of the group for the FA in the UF, we found borderline significant effects for both variables (P = 0.07 for λ1 and P = 0.09 for λ3) (**Tables S1, S2, S3, S4, S5, S6**).

These findings suggest that a combination of increases in axial, and reductions in radial, diffusivity may be contributing to the increased FA detected in the UF of individuals with CD, relative to healthy controls. For a complete report of λ1, λ2, λ3 eigenvalues and ADC measures and for the full ANCOVA analyses of the FA, λ1, λ2, λ3 eigenvalues and ADC see **Tables S1, S2, S3, S4, S5, S6**.

## Discussion

Our study demonstrates that male adolescents with CD, relative to healthy controls, showed increased FA values within the uncinate fascicle (UF), a major bundle of fibers connecting the amygdala and the anterior temporal lobe with the OFC. Of note, a similar result was recently obtained in a different sample of CD youths by an independent research group that employed similar DTI acquisition parameters and tractography analysis methods [25].

However, the present study differs from that performed by Sarkar and colleagues [25], in that we also explored FA differences between groups using whole-brain analyses. This method showed that voxel-wise effects were restricted to a cluster within the external capsule, an area comprising fibers from both the UF and IFOF. Follow-up tractographic analyses demonstrated that white-matter abnormalities were specific to the UF and did not extend to the IFOF. Additional analyses showed that similar results were observed after factoring out individual differences in ADHD symptoms. Our use of complementary methods of analysing DTI data provides cross-validation of the principal finding that FA values are elevated in the UF of adolescents with CD. In contrast to Sarkar et al. [25], our study also focused specifically on the childhood-onset subtype of CD and was restricted to males only, as it is well-established that there are sex differences in white-matter microstructural integrity and trajectories of white-matter development [36].

**Table 2:** Mean fractional anisotropy (FA) values and number of streamlines reconstructed in the uncinate fascicle and inferior frontal occipital fascicle of individuals with CD and healthy controls.

CONDUCT DISORDER (CD)	FA mean value	SD	Mean number of streamlines	SD
Right Uncinate Fascicle	0.44	0.03	68.5	38.6
Left Uncinate Fascicle	0.45	0.04	77.6	37.5
Right Inferior Frontal Occipital Fascicle	0.52	0.03	86.4	48.0
Left Inferior Frontal Occipital Fascicle	0.52	0.03	105.3	67
**CONTROLS**	**FA mean value**	**SD**	**Mean number of streamlines**	**SD**
Right Uncinate Fascicle	0.39	0.04	81.7	43.6
Left Uncinate Fascicle	0.41	0.02	48.0	35.4
Right Inferior Frontal Occipital Fascicle	0.52	0.04	138.9	87.0
Left Inferior Frontal Occipital Fascicle	0.54	0.03	139.9	115.1

It should be noted that another recent study using tract-based spatial statistics (TBSS) analyses to investigate for FA differences across the whole-brain found no FA differences when comparing youths with CD or oppositional defiant disorder and psychopathic traits relative to healthy controls [37]. It is likely that the inconsistencies across studies are due to the use of different methodological approaches, as well as differences in the demographic and clinical features of the respective samples (e.g., the participants’ mean age was ∼15 years in the study by Finger et al., whereas it was ∼18 years in the present study).

As discussed in the Introduction, previous studies have shown reduced gray matter volume in the amygdala and prefrontal cortex of male adolescents with CD [19]–[20], [38]. Our current results show that the anatomical connections between these structures are also abnormal in individuals with CD. Whether this abnormal UF structure is the cause or consequence of the atypical amygdala and prefrontal cortex structure or function remains to be determined. It would also be of interest to characterize the neurocognitive correlates of these alterations in white-matter microstructural integrity.

The precise functions of the UF remain unclear but may involve mediating emotion regulation and social cognitive processes [39]. It is well-established that there is a consistent rise in FA values in white-matter fiber tracts over the first two decades of life [40]–[45]. The longest period of maturation and plasticity is for the uncinate fascicle, which shows increasing FA values throughout the second decade followed by decreasing FA values from around the middle of the third decade [42], [46]. The UF is particularly vulnerable during infancy and early childhood, with premature birth and early social deprivation both associated with lower FA in white-matter pathways many years later [47]–[48]. The current findings of increased FA in CD are therefore unlikely to reflect a pathological insult to the brain in early life. Rather, we suggest that our finding of increased FA in the uncinate fascicle in adolescents with CD is more consistent with the notion of dysfunctional maturation. Specifically, given evidence that white-matter microstructure increases during development, greater FA in the UF of CD individuals (16 to 20 years of age) relative to matched controls may reflect accelerated abnormal maturation of this white-matter pathway. We speculate that this abnormality in adolescents with CD is neurodevelopmental and partly genetic in origin. It may also contribute to core deficits in emotion regulation processes [6]. For example, given the pivotal role of the amygdala and the OFC in emotional behavior [49]–[51], abnormalities in the anatomical pathway linking these regions are likely to result in functional disturbances, including difficulties in regulating emotions during social interactions involving conflict [52].

We note that the opposite pattern of findings has been observed in male adults with antisocial personality disorder or psychopathy [23]–[24], [53], who are reported to show reduced FA values specifically in the UF. Individuals with CD, including those in the current study, tend to score higher on measures of psychopathic traits than controls, and a significant number will go on to satisfy the criteria for antisocial personality disorder or psychopathy in adulthood. We cannot discount that differences between the findings of our study and those of the studies performed using adults may be a function of a number of experimental factors including sample differences, imaging methods, and data analytic procedures. However, accelerated abnormal maturation could also explain the *reduced* FA values in the same white-matter tract found in adult psychopaths aged approximately 35 years of age [23]–[24]. In the typical brain, white-matter microstructural integrity decreases after age 30 (i.e., the FA values of the UF with increasing age follow an inverted U function in healthy individuals). However, this decrease could start earlier following a period of accelerated maturation. In the absence of longitudinal data tracking the development of the UF in individuals with antisocial behavior from childhood to 30 and/or 40 years of age, this explanation is speculative, but could form a testable hypothesis of future longitudinal studies.

As discussed above, FA is an indirect measure of the microstructural integrity of white-matter tracts. Increased FA could result from several different factors including more ‘orderly’ oriented axonal membranes, more compact myelin sheaths parallel to the main fiber direction, or less marked branching or crossing of fibers which would produce a decrease of connections diverging from the main pathway direction [54]–[57]. The analyses of the eigenvalues that specifically reflect axial and radial diffusivity showed that a combination of both measures may contribute to the increased FA found in individuals with CD relative to controls.

Although our current results document a selective abnormality of the UF in adolescents with CD, additional studies in larger samples are required to confirm these results and investigate whether additional white-matter tracts contribute to the pathophysiology of CD. While our sample size was relatively modest for a DTI study, we note that it is comparable to other studies in this area (n = 9 in each group in Craig et al. [23], n = 13 in each group in Motzkin et al. [24], and n = 15 in each group in Sundram et al. [53]). We also acknowledge that the cross-sectional nature of the present study limits the inferences that can be drawn about the neurodevelopmental origins of the FA abnormalities in the UF. Similarly, we cannot infer a causal relationship between white-matter abnormalities and antisocial behavior. As discussed above, a longitudinal study would be particularly beneficial in understanding the normal and abnormal developmental trajectories of white-matter maturation in healthy individuals and in those with CD, and in relating these trajectories to differences in antisocial or aggressive behavior.

In other psychiatric conditions, white matter abnormalities are increasingly reported across the lifespan. For example, schizophrenia is associated with abnormally low FA in widespread circuitry indicative of general connectivity disruptions [58]. Lower FA has also been reported in individuals with unipolar depression and their first degree relatives [59]–[60]. In contrast, recent findings in bipolar disorder indicate increased FA in euthymic individuals [61] with a further study showing concurrent increased *and* reduced FA in different tracts within the same patients suggesting that asymmetry in white matter microstructure may be of importance in the liability for affective disorders [62]. There is also evidence that FA levels are tightly correlated between parents and offspring, suggesting genetic effects on the emergence of abnormal white matter fibers and tract abnormalities in some psychiatric disorders [63]. Of relevance to the present study, ADHD has been associated with elevated FA bilaterally in temporal white-matter structures, but reduced FA in orbitomedial prefrontal white-matter and in the right anterior cingulate bundle [58], [64], also suggesting a potential asymmetry in white-matter structures for this condition. Importantly, however, our primary group effects survived factoring out individual differences in ADHD, suggesting that this factor alone cannot account for the increased FA in the UF of adolescents with CD.

In conclusion, this study provides initial evidence that male adolescents and young adults with CD show increased FA within the uncinate fascicle which connects the amygdala and the orbitofrontal cortex. This finding was obtained using two different, but complementary, methods of analyzing DTI data, and remained significant when controlling for comorbid ADHD symptoms. The uncinate fascicle is implicated in emotion regulation processes, thus abnormal maturation of this white-matter tract in CD may have functional and behavioral consequences. Our results highlight the need for further research exploring maturational schedules across different brain regions and white-matter areas in individuals with CD from childhood into adolescence and adulthood. Childhood and adolescence are critical periods for brain development, hence a detailed understanding of cortical cytoarchitectonic changes and white-matter rearrangements that occur during this time could shed new light on the neurobiological mechanisms that render some individuals especially vulnerable to developing severe antisocial behavior.

## Supporting Information

Table S1 Mean apparent diffusion coefficient (ADC) and λ1 (axial diffusivity), λ2, λ3 (radial diffusivity) eigenvalues in the uncinate fascicle (UF) and inferior frontal-occipital fascicle (IFOF) of male adolescents with Conduct Disorder (CD) and healthy controls.
(DOC)

Table S2 Analyses of Covariance (ANCOVA) results for fractional anisotropy (FA) when including subject-specific region of interest volume (number of voxels, VOX) of each tract and lifetime/ever attention/deficit hyperactivity disorder (ADHD) symptoms as covariates of no interest.
(DOC)

Table S3 Analyses of Covariance (ANCOVA) results for eigenvalue λ1 when including subject-specific region of interest volume (number of voxels, VOX) of each tract and lifetime/ever attention/deficit hyperactivity disorder (ADHD) symptoms as covariates of no interest.
(DOC)

Table S4 Analyses of Covariance (ANCOVA) results for eigenvalue λ2 when including subject-specific region of interest volume (number of voxels, VOX) of each tract and lifetime/ever attention/deficit hyperactivity disorder (ADHD) symptoms as covariates of no interest.
(DOC)

Table S5 Analyses of Covariance (ANCOVA) results for eigenvalue λ3 when including subject-specific region of interest volume (number of voxels, VOX) of each tract and lifetime/ever attention/deficit hyperactivity disorder (ADHD) symptoms as covariates of no interest.
(DOC)

Table S6 Analyses of Covariance (ANCOVA) results for apparent diffusion coefficient (ADC) when including subject-specific region of interest volume (number of voxels, VOX) of each tract and lifetime/ever attention/deficit hyperactivity disorder (ADHD) symptoms as covariates of no interest.
(DOC)
