# Supplementary material for: Abnormal Anatomical Connectivity between the Amygdala and Orbitofrontal Cortex in Conduct Disorder
Source: PLoS One. 2012 Nov 7;7(11):e48789. doi: 10.1371/journal.pone.0048789 (PMC3492256; doi:10.1371/journal.pone.0048789)
Supplement: Table S1 — Mean apparent diffusion coefficient (ADC) and λ1 (axial diffusivity), λ2, λ3 (radial diffusivity) eigenvalues in the uncinate fascicle (UF) and inferior frontal-occipital fascicle (IFOF) of male adolescents with Conduct Disorder (CD) and healthy controls. (DOC) [file pone.0048789.s001.doc]

**Table S1**. Mean apparent diffusion coefficient (ADC) and λ1 (axial diffusivity), λ2, λ3 (radial diffusivity) eigenvalues in the uncinate fascicle (UF) and inferior frontal-occipital fascicle (IFOF) of male adolescents with Conduct Disorder (CD) and healthy controls.

| **CD** | **ADC** | **SD** | **λ1** | **SD** | **λ2** | **SD** | **λ3** | **SD** |
| --- | --- | --- | --- | --- | --- | --- | --- | --- |
| Right UF | 772.72 | 26.73 | 0.001178 | 0.000030 | 0.000659 | 0.000040 | 0.000482 | 0.000039 |
| Left UF | 773.01 | 37.96 | 0.001185 | 0.000031 | 0.001086 | 0.001545 | 0.000479 | 0.000048 |
| Right IFOF | 798.16 | 42.13 | 0.001312 | 0.000065 | 0.000645 | 0.000036 | 0.000439 | 0.000040 |
| Left IFOF | 801.97 | 41.63 | 0.001315 | 0.000045 | 0.000651 | 0.000049 | 0.000442 | 0.000044 |
| **CONTROLS** | **ADC** | **SD** | **λ1** | **SD** | **λ2** | **SD** | **λ3** | **SD** |
| Right UF | 796.75 | 54.84 | 0.001166 | 0.000048 | 0.000710 | 0.000070 | 0.000528 | 0.000061 |
| Left UF | 756.57 | 59.12 | 0.001135 | 0.000066 | 0.000656 | 0.000069 | 0.000483 | 0.000062 |
| Right IFOF | 800.54 | 38.37 | 0.001308 | 0.000054 | 0.000659 | 0.000055 | 0.000437 | 0.000053 |
| Left IFOF | 784.71 | 43.00 | 0.001296 | 0.000069 | 0.000624 | 0.000042 | 0.000417 | 0.000037 |
