# Supplementary material for: Abnormal Anatomical Connectivity between the Amygdala and Orbitofrontal Cortex in Conduct Disorder
Source: PLoS One. 2012 Nov 7;7(11):e48789. doi: 10.1371/journal.pone.0048789 (PMC3492256; doi:10.1371/journal.pone.0048789)
Supplement: Table S2 — Analyses of Covariance (ANCOVA) results for fractional anisotropy (FA) when including subject-specific region of interest volume (number of voxels, VOX) of each tract and lifetime/ever attention/deficit hyperactivity disorder (ADHD) symptoms as covariates of no interest. (DOC) [file pone.0048789.s002.doc]

**Table S2.** Analyses of Covariance (ANCOVA) results for fractional anisotropy (FA) when including subject-specific region of interest volume (number of voxels, VOX) of each tract and lifetime/ever attention-deficit/ hyperactivity disorder (ADHD) symptoms as covariates of no interest.

| **Metric** | **Brain bundles** | **Covariate(s)** | **Effect** | **F statistic** | **d.f.** | **P-value** |
| --- | --- | --- | --- | --- | --- | --- |
| FA | All (IFOF and UF) | VOX | GROUP | 5.89 | 1,23 | 0.023 |
| FA | All (IFOF and UF) | VOX,+/-ADHD | GROUP | 0.58 | 1,22 | 0.314 |
| FA | All (IFOF and UF) | VOX,+/-ADHD | TRACT | 0.31 | 1,23 | 0.583 |
| FA | All (IFOF and UF) | VOX,+/-ADHD | GROUP x TRACT | 8.67 | 1,23 | 0.007 |
| FA | All (IFOF and UF) | VOX,+/-ADHD | HEMISPHERE | 9.94 | 1,23 | 0.004 |
| FA | All (IFOF and UF) | VOX,+/-ADHD | GROUP x HEMISPHERE | 2.23 | 1,23 | 0.149 |
| FA | All (IFOF and UF) | VOX,+/-ADHD | TRACT x HEMISPHERE | 0.10 | 1,23 | 0.749 |
| FA | All (IFOF and UF) | VOX,+/-ADHD | GROUP x TRACT x HEMISPHERE | 0.18 | 1,23 | 0.679 |
|  |  |  |  |  |  |  |
| FA | IFOF | VOX | GROUP | 0.16 | 1,23 | 0.694 |
| FA | IFOF | VOX,+/-ADHD | GROUP | 0.70 | 1,22 | 0.411 |
| FA | IFOF | VOX,+/-ADHD | HEMISPHERE | 5.62 | 1,23 | 0.026 |
| FA | IFOF | VOX,+/-ADHD | GROUP x HEMISPHERE | 2.47 | 1,23 | 0.130 |
|  |  |  |  |  |  |  |
| FA | UF | VOX | GROUP | 20.03 | 1,23 | 0.0002 |
| FA | UF | VOX,+/-ADHD | GROUP | 5.11 | 1,22 | 0.034 |
| FA | UF | VOX,+/-ADHD | HEMISPHERE | 5.83 | 1,23 | 0.024 |
| FA | UF | VOX,+/-ADHD | GROUP x HEMISPHERE | 0.93 | 1,23 | 0.344 |

Key: +/-ADHD, factoring out lifetime/ever ADHD symptoms; IFOF, inferior frontal-occipital fascicle; UF, uncinate fascicle; d.f., degrees of freedom
