# Supplementary material for: Abnormal Anatomical Connectivity between the Amygdala and Orbitofrontal Cortex in Conduct Disorder
Source: PLoS One. 2012 Nov 7;7(11):e48789. doi: 10.1371/journal.pone.0048789 (PMC3492256; doi:10.1371/journal.pone.0048789)
Supplement: Table S3 — Analyses of Covariance (ANCOVA) results for eigenvalue λ1 when including subject-specific region of interest volume (number of voxels, VOX) of each tract and lifetime/ever attention/deficit hyperactivity disorder (ADHD) symptoms as covariates of no interest. (DOC) [file pone.0048789.s003.doc]

**Table S3. Analyses of Covariance (ANCOVA) results for eigenvalue λ1 when including subject-specific region of interest volume (number of voxels, VOX) of each tract and lifetime/ever attention-deficit/hyperactivity disorder (ADHD) symptoms as covariates of no interest.**

| **Metric** | **Brain bundles** | **Covariate(s)** | **Effect** | **F statistic** | **d.f.** | **P-value** |
| --- | --- | --- | --- | --- | --- | --- |
| λ1 | All (IFOF and UF) | VOX | GROUP | 1.60 | 1,23 | 0.219 |
| λ1 | All (IFOF and UF) | VOX,+/-ADHD | GROUP | 0.27 | 1,22 | 0.609 |
| λ1 | All (IFOF and UF) | VOX,+/-ADHD | TRACT | 2.49 | 1,23 | 0.128 |
| λ1 | All (IFOF and UF) | VOX,+/-ADHD | GROUP x TRACT | 0.94 | 1,23 | 0.341 |
| λ1 | All (IFOF and UF) | VOX,+/-ADHD | HEMISPHERE | 1.42 | 1,23 | 0.246 |
| λ1 | All (IFOF and UF) | VOX,+/-ADHD | GROUP x HEMISPHERE | 1.73 | 1,23 | 0.201 |
| λ1 | All (IFOF and UF) | VOX,+/-ADHD | TRACT x HEMISPHERE | 0.34 | 1,23 | 0.564 |
| λ1 | All (IFOF and UF) | VOX,+/-ADHD | GROUP x TRACT x HEMISPHERE | 1.03 | 1,23 | 0.320 |
|  |  |  |  |  |  |  |
| λ1 | IFOF | VOX | GROUP | 0.22 | 1,23 | 0.646 |
| λ1 | IFOF | VOX,+/-ADHD | GROUP | 2.00 | 1,22 | 0.172 |
| λ1 | IFOF | VOX,+/-ADHD | HEMISPHERE | 1.42 | 1,23 | 0.390 |
| λ1 | IFOF | VOX,+/-ADHD | GROUP x HEMISPHERE | 2.56 | 1,23 | 0.123 |
|  |  |  |  |  |  |  |
| λ1 | UF | VOX | GROUP | 3.41 | 1,23 | 0.078 |
| λ1 | UF | VOX,+/-ADHD | GROUP | 1.98 | 1,22 | 0.172 |
| λ1 | UF | VOX,+/-ADHD | HEMISPHERE | 0.78 | 1,23 | 0.390 |
| λ1 | UF | VOX,+/-ADHD | GROUP x HEMISPHERE | 2.56 | 1,23 | 0.123 |

Key: λ1, eigenvalues (axial diffusivity); +/-ADHD, factoring out lifetime/ever ADHD symptoms; IFOF, inferior frontal-occipital fascicle; UF, uncinate fascicle; d.f., degrees of freedom
