# Supplementary material for: Abnormal Anatomical Connectivity between the Amygdala and Orbitofrontal Cortex in Conduct Disorder
Source: PLoS One. 2012 Nov 7;7(11):e48789. doi: 10.1371/journal.pone.0048789 (PMC3492256; doi:10.1371/journal.pone.0048789)
Supplement: Table S4 — Analyses of Covariance (ANCOVA) results for eigenvalue λ2 when including subject-specific region of interest volume (number of voxels, VOX) of each tract and lifetime/ever attention/deficit hyperactivity disorder (ADHD) symptoms as covariates of no interest. (DOC) [file pone.0048789.s004.doc]

**Table S4. Analyses of Covariance (ANCOVA) results for eigenvalue λ2 when including subject-specific region of interest volume (number of voxels, VOX) of each tract and lifetime/ever attention-deficit/hyperactivity disorder (ADHD) symptoms as covariates of no interest.**

| **Metric** | **Brain bundles** | **Covariate(s)** | **Effect** | **F statistic** | **d.f.** | **P-value** |
| --- | --- | --- | --- | --- | --- | --- |
| λ2 | All (IFOF and UF) | VOX | GROUP | 0.64 | 1,23 | 0.432 |
| λ2 | All (IFOF and UF) | VOX,+/-ADHD | GROUP | 0.18 | 1,22 | 0.676 |
| λ2 | All (IFOF and UF) | VOX,+/-ADHD | TRACT | 3.18 | 1,23 | 0.688 |
| λ2 | All (IFOF and UF) | VOX,+/-ADHD | GROUP x TRACT | 0.56 | 1,23 | 0.463 |
| λ2 | All (IFOF and UF) | VOX,+/-ADHD | HEMISPHERE | 0.38 | 1,23 | 0.544 |
| λ2 | All (IFOF and UF) | VOX,+/-ADHD | GROUP x HEMISPHERE | 1.42 | 1,23 | 0.245 |
| λ2 | All (IFOF and UF) | VOX,+/-ADHD | TRACT x HEMISPHERE | 0.79 | 1,23 | 0.384 |
| λ2 | All (IFOF and UF) | VOX,+/-ADHD | GROUP x TRACT x HEMISPHERE | 0.94 | 1,23 | 0.342 |
|  |  |  |  |  |  |  |
| λ2 | IFOF | VOX | GROUP | 0.03 | 1,23 | 0.865 |
| λ2 | IFOF | VOX,+ADHD | GROUP | 0.01 | 1,22 | 0.944 |
| λ2 | IFOF | VOX,+/-ADHD | HEMISPHERE | 1.47 | 1,23 | 0.238 |
| λ2 | IFOF | VOX,+/-ADHD | GROUP x HEMISPHERE | 4.43 | 1,23 | 0.046 |
|  |  |  |  |  |  |  |
| λ2 | UF | VOX | GROUP | 1.58 | 1,23 | 0.221 |
| λ2 | UF | VOX,+/-ADHD | GROUP | 1.11 | 1,22 | 0.303 |
| λ2 | UF | VOX,+/-ADHD | HEMISPHERE | 0.49 | 1,23 | 0.490 |
| λ2 | UF | VOX,+/-ADHD | GROUP x HEMISPHERE | 1.94 | 1,23 | 0.176 |

Key: λ2, eigenvalues (radial diffusivity); +/-ADHD, factoring out lifetime/ever ADHD symptoms; IFOF, inferior frontal-occipital fascicle; UF, uncinate fascicle; d.f., degrees of freedom
