# Supplementary material for: Abnormal Anatomical Connectivity between the Amygdala and Orbitofrontal Cortex in Conduct Disorder
Source: PLoS One. 2012 Nov 7;7(11):e48789. doi: 10.1371/journal.pone.0048789 (PMC3492256; doi:10.1371/journal.pone.0048789)
Supplement: Table S5 — Analyses of Covariance (ANCOVA) results for eigenvalue λ3 when including subject-specific region of interest volume (number of voxels, VOX) of each tract and lifetime/ever attention/deficit hyperactivity disorder (ADHD) symptoms as covariates of no interest. (DOC) [file pone.0048789.s005.doc]

**Table S5.** Analyses of Covariance (ANCOVA) results for eigenvalue λ3 when including subject-specific region of interest volume (number of voxels, VOX) of each tract and lifetime/ever attention-deficit/hyperactivity disorder (ADHD) symptoms as covariates of no interest.

| **Metric** | **Brain bundles** | **Covariate(s)** | **Effect** | **F statistic** | **d.f.** | **P-value** |
| --- | --- | --- | --- | --- | --- | --- |
| λ3 | All (IFOF and UF) | VOX | GROUP | 0.41 | 1,23 | 0.527 |
| λ3 | All (IFOF and UF) | VOX+/-ADHD | GROUP | 0.07 | 1,22 | 0.793 |
| λ3 | All (IFOF and UF) | VOX+/-ADHD | TRACT | 2.41 | 1,23 | 0.134 |
| λ3 | All (IFOF and UF) | VOX+/-ADHD | GROUP x TRACT | 2.49 | 1,23 | 0.129 |
| λ3 | All (IFOF and UF) | VOX+/-ADHD | HEMISPHERE | 8.68 | 1,23 | 0.007 |
| λ3 | All (IFOF and UF) | VOX+/-ADHD | GROUP x HEMISPHERE | 7.83 | 1,23 | 0.013 |
| λ3 | All (IFOF and UF) | VOX+/-ADHD | TRACT x HEMISPHERE | 1.17 | 1,23 | 0.289 |
| λ3 | All (IFOF and UF) | VOX+/-ADHD | GROUP x TRACT x HEMISPHERE | 0.20 | 1,23 | 0.659 |
|  |  |  |  |  |  |  |
| λ3 | IFOF | VOX | GROUP | 0.45 | 1,23 | 0.508 |
| λ3 | IFOF | VOX+/-ADHD | GROUP | 0.50 | 1,22 | 0.486 |
| λ3 | IFOF | VOX+/-ADHD | HEMISPHERE | 1.20 | 1,23 | 0.286 |
| λ3 | IFOF | VOX+/-ADHD | GROUP x HEMISPHERE | 2.40 | 1,23 | 0.134 |
|  |  |  |  |  |  |  |
| λ3 | UF | VOX | GROUP | 2.98 | 1,23 | 0.098 |
| λ3 | UF | VOX+/-ADHD | GROUP | 0.36 | 1,22 | 0.562 |
| λ3 | UF | VOX+/-ADHD | HEMISPHERE | 3.84 | 1,23 | 0.062 |
| λ3 | UF | VOX+/-ADHD | GROUP x HEMISPHERE | 2.94 | 1,23 | 0.101 |

Key: λ3, eigenvalues (radial diffusivity); +/-ADHD, factoring out lifetime/ever ADHD symptoms; IFOF, inferior frontal-occipital fascicle; UF, uncinate fascicle; d.f., degrees of freedom
