# Supplementary material for: Abnormal Anatomical Connectivity between the Amygdala and Orbitofrontal Cortex in Conduct Disorder
Source: PLoS One. 2012 Nov 7;7(11):e48789. doi: 10.1371/journal.pone.0048789 (PMC3492256; doi:10.1371/journal.pone.0048789)
Supplement: Table S6 — Analyses of Covariance (ANCOVA) results for apparent diffusion coefficient (ADC) when including subject-specific region of interest volume (number of voxels, VOX) of each tract and lifetime/ever attention/deficit hyperactivity disorder (ADHD) symptoms as covariates of no interest. (DOC) [file pone.0048789.s006.doc]

**Table S6. Analyses of Covariance (ANCOVA) results for ADC when including subject-specific region of interest volume (number of voxels, VOX) of each tract and lifetime/ever attention-deficit/hyperactivity disorder (ADHD) symptoms as covariates of no interest.**

| **Metric** | **Brain bundles** | **Covariate(s)** | **Effect** | **F statistic** | **d.f.** | **P-value** |
| --- | --- | --- | --- | --- | --- | --- |
| ADC | All (IFOF and UF) | VOX | GROUP | 0.02 | 1,23 | 0.884 |
| ADC | All (IFOF and UF) | VOX,+ADHD | GROUP | 0.04 | 1,22 | 0.840 |
| ADC | All (IFOF and UF) | VOX,+/-ADHD | TRACT | 10.27 | 1,23 | 0.004 |
| ADC | All (IFOF and UF) | VOX,+/-ADHD | GROUP x TRACT | 0.28 | 1,23 | 0.600 |
| ADC | All (IFOF and UF) | VOX,+/-ADHD | HEMISPHERE | 4.69 | 1,23 | 0.040 |
| ADC | All (IFOF and UF) | VOX,+/-ADHD | GROUP x HEMISPHERE | 4.89 | 1,23 | 0.037 |
| ADC | All (IFOF and UF) | VOX,+/-ADHD | TRACT x HEMISPHERE | 1.35 | 1,23 | 0.238 |
| ADC | All (IFOF and UF) | VOX,+/-ADHD | GROUP x TRACT x HEMISPHERE | 0.39 | 1,23 | 0.537 |
|  |  |  |  |  |  |  |
| ADC | IFOF | VOX | GROUP | 0.05 | 1,23 | 0.827 |
| ADC | IFOF | VOX,+ADHD | GROUP | 0.01 | 1,22 | 0.936 |
| ADC | IFOF | VOX,+/-ADHD | HEMISPHERE | 0.52 | 1,23 | 0.480 |
| ADC | IFOF | VOX,+/-ADHD | GROUP x HEMISPHERE | 1.22 | 1,23 | 0.280 |
|  |  |  |  |  |  |  |
| ADC | UF | VOX | GROUP | 0.39 | 1,23 | 0.540 |
| ADC | UF | VOX,+ADHD | GROUP | 0.02 | 1,22 | 0.894 |
| ADC | UF | VOX,+/-ADHD | HEMISPHERE | 2.94 | 1,23 | 0.100 |
| ADC | UF | VOX,+/-ADHD | GROUP x HEMISPHERE | 3.18 | 1,23 | 0.088 |

Key: ADC, apparent diffusion coefficient; +/-ADHD, factoring out lifetime/ever ADHD symptoms; IFOF, inferior frontal-occipital fascicle; UF, uncinate fascicle; d.f., degrees of freedom
